# A Diagnostic Pitfall: Retrocaval Nerve Sheath Tumor

**DOI:** 10.7759/cureus.95435

**Published:** 2025-10-26

**Authors:** On Wa Ng, Fung Him Ng, Yuen Fun Mak

**Affiliations:** 1 Diagnostic and Interventional Radiology Department, Princess Margaret Hospital, Kowloon West Cluster, Hong Kong, HKG; 2 Pathology, Princess Margaret Hospital, Hong Kong, HKG

**Keywords:** lymph nodes, nerve sheath neoplasms, retrocaval mass, retroperitoneal neoplasms, spindle cell neoplasm

## Abstract

A 78-year-old female with a history of resected colorectal adenocarcinoma presented with progressively rising serum carcinoembryonic antigen (CEA) levels since 2022. An August 2023Positron Emission Tomography/Computed Tomography (PET-CT) revealed a 1.4 cm fluorodeoxyglucose (FDG)-avid retrocaval lesion, initially raising suspicion for metastatic lymphadenopathy given her cancer history. Despite receiving stereotactic body radiotherapy and remaining asymptomatic, her CEA levels continued to climb, and the lesion mildly enlarged to 1.8 cm by November 2024. This persistent elevation and lesion growth necessitated a definitive tissue diagnosis. Subsequent biopsy showed a spindle cell neoplasm. Immunohistochemical staining, crucial for characterizing soft-tissue tumor differentiation, revealed diffuse positivity for SOX10 and S100 protein, consistent with a primary nerve sheath tumor rather than metastatic disease. This case highlights the diagnostic challenge posed by FDG-avid lesions in patients with a history of malignancy, underscoring the importance of considering rare differential diagnoses and pursuing tissue diagnosis when imaging is equivocal or clinical presentation deviates from expected metastatic behavior.

## Introduction

Accurate interpretation of imaging findings in oncology patients is critical, especially when evaluating new or evolving lesions in the context of rising tumor markers. Carcinoembryonic antigen (CEA) is a commonly used biomarker for surveillance in colorectal cancer (CRC), and elevated levels often prompt investigations for recurrent or metastatic disease [1,2].

18F-fluorodeoxyglucose (FDG) Positron Emission Tomography/Computed Tomography (PET/CT) is highly sensitive for detecting occult CRC recurrence in this setting, with sensitivity reported as high as 97% [2,3]. However, the radiotracer FDG is not cancer-specific, and not all FDG-avid lesions indicate malignancy recurrence, posing a diagnostic challenge due to false-positive results from benign and inflammatory processes [4,5].

Retroperitoneal and retrocaval masses are uncommon and encompass a broad differential diagnosis, ranging from nodal metastases to primary soft-tissue tumors [6,7]. Misinterpretation may lead to inappropriate management, highlighting the necessity of histological confirmation in many cases to prevent unnecessary or delayed treatment [7].

## Case presentation

**Figure 1:**
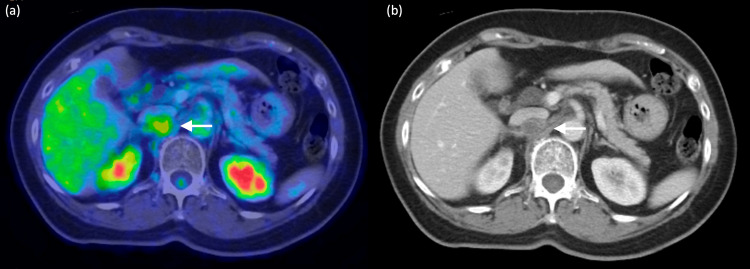
(a) FDG PET Scan. The retrocaval lesion showing FDG avidity (white arrow). (b) CT Scan: A retrocaval hypodense ovoid lesion (white arrow), demonstrating hypoenhancement on the portovenous phase.

A 78-year-old female, with a history of pT1N0 moderately differentiated adenocarcinoma of the rectosigmoid colon treated with anterior resection in May 2006, presented with a progressive rise in serum carcinoembryonic antigen (CEA) levels since 2022. In August 2023, to investigate the elevated CEA, an 18F-fluorodeoxyglucose (FDG) PET-CT scan was performed. This scan revealed a 1.4 cm FDG-avid retrocaval lesion with a maximum standardized uptake value (SUVmax) of 4.8, compared to a background liver SUVmax of 3.3 (Figure 1). Given the patient’s history of colorectal malignancy, the moderate FDG avidity initially raised suspicion for metastatic lymphadenopathy.

**Figure 2:**
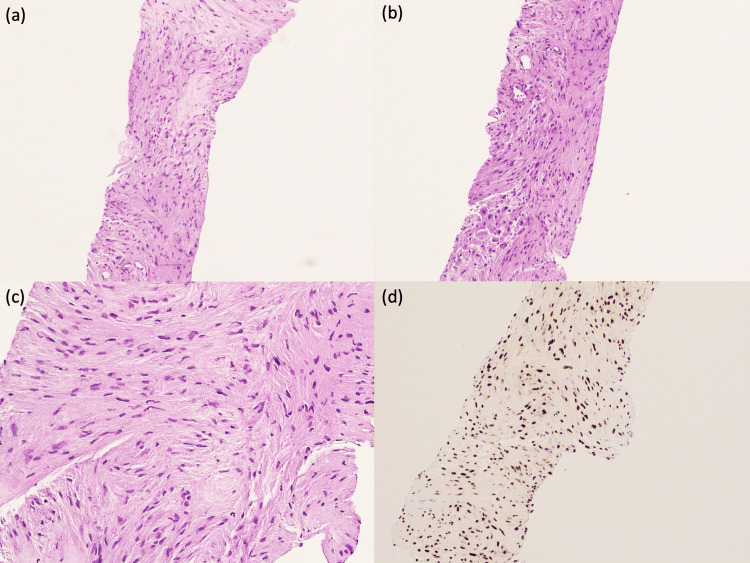
H&E stain. Low-power view (a,b) shows loosely arranged spindle cells in collagenous stroma. High-power view (c) reveals spindle cells with dark, wavy nuclei and scanty cytoplasm; nuclear atypia is mild. (d) Immunohistochemical staining for SOX10 demonstrates diffuse nuclear positivity within the tumor cells.

Despite the patient remaining asymptomatic and repeated colonoscopies showing no evidence of local recurrence or new mucosal lesions, her CEA levels continued to rise. In November 2023, she underwent stereotactic body radiotherapy (SBRT) at a private facility. However, follow-up CEA levels continued to rise. A subsequent CT scan in November 2024 showed mild enlargement of the lesion to 1.8 cm (from 1.4 cm in 2023). The persistence and enlargement of the lesion despite radiotherapy, coupled with the continued elevation in CEA levels, prompted the need for a definitive tissue diagnosis. A biopsy was performed in March 2025. Histological examination of the retrocaval lesion revealed loosely arranged spindle cells with dark staining, wavy nuclei, and inconspicuous cytoplasm, set within a collagenous background. There is no significant nuclear atypia, and mitosis is inconspicuous. Features are consistent with a spindle cell neoplasm. Immunohistochemical staining showed diffuse positivity for SOX10 and S100 protein, while it was negative for AE1/AE3, CD34, calretinin, desmin, CDK4, and GFAP markers (Figure 2). These findings were consistent with a primary nerve sheath tumor, rather than metastatic disease [8,9].

## Discussion

Retroperitoneal nerve sheath tumors are a rare group of neoplasms, posing a diagnostic and therapeutic challenge due to their non-specific presentation and location within the deep retroperitoneal space. Benign nerve sheath tumors, primarily schwannomas and less frequently neurofibromas, comprise approximately 5% of all retroperitoneal tumors [8]. Given the compliance of the retroperitoneum, these typically slow-growing tumors are commonly found incidentally and often reach a considerable size before presenting with symptoms, such as abdominal pain, a palpable mass, or, rarely, compressive symptoms like nerve or venous compression [8,9]. In contrast, malignant peripheral nerve sheath tumors (MPNSTs) in the retroperitoneum are extremely rare, representing only about 1% of all MPNSTs [10]. Accurate preoperative differentiation between benign and malignant entities is critical for proper surgical planning, as MPNSTs are highly aggressive with a poor prognosis [11].

Cross-sectional imaging, including CT and MRI, is essential for initial characterization, though specific features can overlap with other retroperitoneal masses [8]. On CT, benign schwannomas typically appear as well-circumscribed ovoid or spherical masses, while cystic changes are more common in larger lesions [8]. MRI offers superior soft-tissue contrast, with schwannomas typically demonstrating T1 hypointense and T2 hyperintense signal. Mixed T1 and T2 signals can be seen in hemorrhagic and cystic changes, while the solid component often shows heterogeneous or peripheral gadolinium contrast enhancement [8]. However, definitive diagnosis requires tissue sampling. Histologically, classic schwannomas exhibit alternating hypercellular Antoni A areas and hypocellular myxoid Antoni B areas [12].

Immunohistochemistry (IHC) is essential for confirming neural origin and determining malignancy. Both benign schwannomas and neurofibromas typically exhibit diffuse, strong positivity for S100 protein and strong nuclear SOX10 reactivity [12,13]. SOX10 is generally considered a highly specific neural crest marker [13]. In contrast, MPNSTs show reduced sensitivity for these markers, often displaying only focal S100 expression or only partial/focal SOX10 expression [14,15]. Notably, loss of trimethylation of lysine 27 of histone H3 (H3K27me3) on immunohistochemistry is observed in 60-80% of high-grade cases and is relatively specific for MPNST [16]. A high Ki-67 labeling index is a strong indicator of malignancy and is crucial for distinguishing aggressive tumors like cellular schwannomas from true MPNSTs. Higher Ki-67 levels are also correlated with poorer survival rates in MPNSTs [17].

Complete surgical excision with negative margins remains the primary treatment for both benign and malignant retroperitoneal nerve sheath tumors [9]. For benign schwannomas, complete resection is typically curative, with recurrence being rare [9]. For MPNSTs, the prognosis is generally poor, even after curative-intent surgery, due to high rates of local recurrence and distant metastasis [11]. Neoadjuvant and adjuvant radiotherapy may be employed for MPNSTs to improve local control and to make surgery possible, but the role of chemotherapy remains limited [18,19].

## Conclusions

This case illustrates a significant diagnostic challenge. The presence of an FDG-avid retrocaval lesion in a patient with a history of CRC and rising CEA levels strongly mimics metastatic disease. However, the atypical imaging features and the final histopathological and immunohistochemical results revealed a primary nerve sheath tumor in the retroperitoneum. Distinguishing between these conditions based on imaging alone can be difficult; this case underscores the importance of considering rare differential diagnoses and pursuing a tissue diagnosis when imaging features are equivocal or when the clinical presentation differs from the expected behavior of metastatic disease.
